# Dipeptidyl peptidase-4 inhibitor gemigliptin protects against vascular calcification in an experimental chronic kidney disease and vascular smooth muscle cells

**DOI:** 10.1371/journal.pone.0180393

**Published:** 2017-07-07

**Authors:** Soon-Youn Choi, Hye-Myung Ryu, Eun-Joo Oh, Ji-Young Choi, Jang-Hee Cho, Chan-Duck Kim, Yong-Lim Kim, Sun-Hee Park

**Affiliations:** 1 Division of Nephrology and Department of Internal Medicine, Kyungpook National University School of Medicine, Daegu, Korea; 2 BK21 Plus Biomedical Convergence Program, Department of Biomedical Science, Kyungpook National University, Daegu, Korea; 3 Cell and Matrix Research Institute, Kyungpook National University, Daegu, Korea; Augusta University, UNITED STATES

## Abstract

Although dipeptidyl peptidase-4 inhibitors, a class of antidiabetic drugs, have various pleiotropic effects, it remains undetermined whether gemigliptin has a beneficial effect on vascular calcification. Therefore, this study was performed to evaluate the effect of gemigliptin on vascular calcification in a rat model of adenine-induced chronic kidney disease and in cultured vascular smooth muscle cells. Gemigliptin attenuated calcification of abdominal aorta and expression of RUNX2 in adenine-induced chronic kidney disease rats. In cultured vascular smooth muscle cells, phosphate-induced increase in calcium content was reduced by gemigliptin. Gemigliptin reduced phosphate-induced *PiT-1* mRNA expression, reactive oxygen species generation, and NADPH oxidase mRNA expression (*p22*^*phox*^ and *NOX4*). The reduction of oxidative stress by gemigliptin was associated with the downregulation of phospho-PI3K/AKT expression. High phosphate increased the expression of frizzled-3 (*FDZ3*) and decreased the expression of dickkopf-related protein-1 (*DKK-1*) in the Wnt pathway. These changes were attenuated by gemigliptin treatment. Gemigliptin restored the decreased expression of vascular smooth muscle cells markers (*α-SMA* and *SM22α*) and increased expression of osteogenic makers (*CBFA1*, *OSX*, *E11*, and *SOST*) induced by phosphate. In conclusion, gemigliptin attenuated vascular calcification and osteogenic trans-differentiation in vascular smooth muscle cells via multiple steps including downregulation of PiT-1 expression and suppression of reactive oxygen species generation, phospho-PI3K/AKT, and the Wnt signaling pathway.

## Introduction

Vascular calcification (VC) occurs more frequently in patients with chronic kidney disease (CKD) and diabetes mellitus (DM), and usually affects blood vessels including the aorta as well as medium- and small-sized vessels such as coronary arteries [1]. It is characterized by accelerated mineral deposition within the medial layer of arteries. VC increases the stiffness of the arterial wall and negatively influences heart function by increasing cardiac afterload and left ventricular hypertrophy, and decreasing coronary blood flow. Clearly, VC has an impact on cardiovascular events and mortality in CKD patients as well as patients with DM [2–4]. Hyperphosphatemia, one of the major abnormalities in CKD-mineral bone disorder (MBD), is primarily associated with VC in patients with kidney disease. Previously, high phosphate-induced VC was reported to indicate passive calcium-phosphate deposition [5]. However, recently VC has been recognized as a highly active process. It is associated with a multifactorial mechanism, which includes calcium/phosphate dysregulation, calciprotein particles, impaired anti-calcific mechanism such as dysfunction of inhibitors, and trans-differentiation of vascular smooth muscle cell (VSMC) phenotype. VSMCs trans-differentiation is characterized by loss of VSMC marker proteins [smooth muscle (SM) α-actin and SM22α] and gain of osteoblast marker proteins [runt-related transcription factor-2 (RUNX2; also called CBFA1), osterix (OSX), osteocalcin (OC), DMP-1, sclerostin (SOST), and E11]. This is a process similar to physiological bone formation [6]. In addition, Wnt signaling has been reported as a main master regulator for activating the expression of osteoblast trans-differentiation markers to induce VC [7]. Wnt proteins bind to the plasma membrane frizzled (FDZ) receptors and low-density lipoprotein receptor-related protein-5/6 (LRP5/6) co-receptor, and regulate downstream signaling by dephosphorylation of β-catenin. Activation of Wnt signaling regulates trans-differentiation of the osteogenic phenotype through the expression of several bone-related proteins such as osterix (OSX), osteocalcin (OC), and sclerostin (SOST) [8].

Dipeptidyl peptidase-4 (DPP-4) inhibitors, novel antidiabetic drugs, have the ability to control blood glucose by inhibiting the degradation of incretin hormones such as type I glucagon-like peptide (GLP-1) and glucose-dependent insulinotropic polypeptide (GIP) without the risk of hypoglycemia or body weight gain [9, 10]. Besides, recent studies have suggested that DPP-4 inhibitors have cardio-protective effects in addition to their glucose-lowering effect in experimental studies [11–13]. The DPP-4 inhibitor des-fluoro-sitagliptin reduces restenosis in the carotid artery following balloon injury in type 2 DM rats [14]. Sitagliptin treatment in ApoE KO mice reduced plaque inflammation through inhibition of monocyte migration and macrophage MMP-9 release [15]. Linagliptin significantly reduced neointima formation in a vascular injury model of non-diabetic mice [13], and in obstructed aortic and endothelial stiffness induced by a western diet in female mice [16]. Cardiovascular pleiotropic actions of DPP-4 inhibitors include reduction of reactive oxygen species (ROS) generation, prevention of mitochondrial depolarization, improvement of endothelial functions, and reduction of vascular inflammation and oxidative stress [11, 12, 17]. However, clinical studies regarding the cardio-protective effect of DPP-4 inhibitors have not shown consistent results. Recently, two randomized clinical trials of alogliptin and saxagliptin did not support the cardiovascular protective effects of DPP-4 inhibitors in type 2 diabetic patients [18, 19].

Gemigliptin, a novel DPP-4 inhibitor, developed in Korea (Zemiglo^®^, LG Life Science, Seoul, South Korea), is a highly competitive and selective DPP-4 inhibitor. Gemigliptin improves glucose tolerance by increasing insulin secretion and decreasing glucagon secretion *in vivo* and *in vitro* [20]. Previous studies have reported on the pleiotropic effect of gemigliptin, besides its glucose lowering effect, including inhibition of LPS-induced pro-inflammatory effects in vascular endothelial cells by attenuating NF-kappaB and JNK signaling via Akt/AMPK-dependent mechanisms [21], and protection against renal interstitial fibrosis in a mouse model of ureteral obstruction [22]. However, there are few studies on the effects of gemigliptin on VC. Therefore, this study was performed to investigate whether gemigliptin attenuates VC in an adenine-induced CKD model and to explore the possible mechanisms by which gemigliptin is involved in this process using cultured VSMCs.

## Materials and methods

### Experimental adenine-induced chronic kidney disease rat model

Twelve-week-old male Sprague-Dawley (SD) rats (380–390 g) were purchased from Samtako Co. Ltd. (Osan, Korea). The animals were housed under standardized conditions (temperature at 20–22°C, humidity at 50–60%, and 12:12 h light/dark cycles) and allowed free access to food and tap water throughout the experiments. The animal study was approved by the Animal Care and Use Committee at the Kyungpook National University (Permit Number: KNU-2014-0099), and all experiments were performed in accordance with the guidelines of the Animal Care and Use Committee of Laboratory Animals of Kyungpook National University. The CKD model was induced by feeding SD rats a 0.75% adenine diet and low protein diet for 4 weeks without any surgical procedure. Previous reports showed that medial calcification of aorta occurs within 4 weeks of the initiation of 0.75% adenine diet, which is more consistent when fed with a low protein diet [23]. Sprague-Dawley (SD) rats were divided into four groups after one week of acclimatization as follows: control group (n = 5; low protein (LP) control group; 2.4% protein (casein) and 75.3% carbohydrate, 4.6% fat, 5% cellulose, 1.06% calcium, and 0.92% phosphorus; TD05030; Harlan, Teklad), adenine group (n = 5; 0.75% adenine, 2.4% protein and 74.5% carbohydrate; TD05031; Harlan, Teklad), adenine-gemigliptin (10 mg/kg) group (n = 6, AG10), and adenine-gemigliptin (20 mg/kg) group (n = 6, AG20), which were fed for 4 weeks. Gemigliptin was injected intraperitoneally once daily at a dose of 10 mg/kg or 20 mg/kg, which was started at the same time as the adenine. Food intake and body weight were checked every week. At the end of the 4 weeks *in vivo* experiments, all animals were sacrificed under anesthesia breathing with 1.5% isoflurane (Hana Pharma Corp., Kyonggi-Do, Korea) via the mask and efforts were made to minimize pain. Serum samples were collected by heart puncture into EDTA/acid-free tubes. After centrifuging at 1,500 *g* for 10 min at 4°C, the serum levels of blood urea nitrogen (BUN), creatinine, calcium, and phosphate were measured at SamKwang Laboratory (Daegu, Korea).

### Assessment of vascular calcification using Von Kossa staining

VC was assessed by Von Kossa’s method. After isolation of abdominal aortic tissues, tissue was fixed with 4% paraformaldehyde (pH 7.4) and embedded in paraffin. Three-micrometer tissue sections were deparaffinized, rehydrated, and incubated with 1% silver nitrate (AgNO_3_; Sigma, St. Louis, MO, USA) under ultraviolet light for 30 min. Then, unreacted silver was removed by treating with 5% sodium thiosulfate (Na_2_S_2_O_3_; Sigma, St. Louis, MO, USA) for 5 min. Nuclei were counterstained with hematoxylin and eosin for 5 min. The percentage of calcified area was calculated as the ratio of the Von Kossa positive area versus the total tissue area using Image J analysis software (NIH, Bethesda, MD). The results were calculated as percentage of control.

### Cell culture and treatment

Human aortic smooth muscle cells were purchased from American Type Culture Collection (ATCC; Manassas, VA, USA). Cells were cultured in Dulbecco’s Modified Eagle’s Medium (DMEM) supplemented with 10% fetal bovine serum, 100 U/mL penicillin, and 100 μg/mL streptomycin at 37°C, 5% CO_2_ conditions. Cells were used between the 5^th^ and 8^th^ passage for the experiments. VSMCs were incubated with 3 mM inorganic phosphate (mixture of Na_2_HPO_4_ and NaH_2_PO_4_, pH 7.4) and/or 50 μM gemigliptin (LG Life Science Ltd., Seoul, South Korea) for the indicated number of days. The medium was exchanged every 2 days.

### Quantification and deposition of calcium

After incubation for 14 days, VSMCs were washed with Dulbecco’s phosphate-buffered saline (D-PBS) and decalcified with 0.6 N HCl for 24h at 37°C to quantify calcium deposition. After centrifuging at 12,000g for 5 min, the calcium content of the supernatant was determined colorimetrically using a QuantiChrom Calcium Assay Kit (BioAssay Systems, Hayward, CA, USA). The calcium content was normalized by the total cellular protein and expressed as percentage of control. Calcium deposition was visualized using alizarin red staining. VSMCs treated for 14 days were washed 2 times with D-PBS, fixed with 4% formaldehyde for 10 min, rinsed 3 times with distilled water, stained with 2% alizarin red staining solution (pH 4.2; Sigma, St. Louis, MO, USA) for 30 min, and rinsed with distilled water to remove excess dye. Mineralized nodules were observed as red deposits.

### Cell viability assay

To evaluate toxicity of phosphate and/or gemigliptin, VSMCs were seeded in 96-well plates and incubated with serum-free medium for 24 h. After starvation, the cells were treated with 3 mM inorganic phosphate and/or 50 μM gemigliptin for 14 days. Treated cells were incubated with 200 μL DMEM containing 500 μg/mL 3-(4, 5-dimethylthiazol-2yl)-2, 5-diphenyltetrazolium bromide (MTT; Amresco, Solon, OH, USA) for 4h. The converted dye was solubilized with 200 μL dimethyl sulfoxide (DMSO; Amresco, Solon, OH, USA) and the absorbance was measured at 570 nm. The value was expressed as percentage of control.

### Intracellular reactive oxygen species measurement

To measure intracellular ROS generation, VSMCs were seeded in 12-well plate and incubated with 3 mM inorganic phosphate and various gemigliptin concentrations (50–200 mM) for 2 h. After treatment, VSMCs were stained with 10 μM 2′, 7′-dichlorodihydrofluorescein diacetate (H_2_DCFDA; Molecular Probes, Eugene, OR, USA) in serum-free medium for 40 min. The stained cells were visualized with fluorescence microscopy (Nikon, Tokyo, Japan). The fluorescence intensity was measured from the cellular lysate after incubation with lysis buffer (0.1% Triton X-100 and 0.5 mM EDTA in D-PBS) at 480 nm excitation and 520 nm emission using a fluorescence microplate reader (Molecular Devices Corp., Silicon Valley, CA, USA). The value was normalized by the total cellular protein and expressed as percentage of control.

### Hydrogen peroxide assay

To check the concentration of H_2_O_2_ in the cells, VSMCs were grown in 96-well black plate and incubated with 3 mM inorganic phosphate and various gemigliptin concentrations (50–200 mM) for 2h. The levels of H_2_O_2_ were measured using the Amplex Red Hydrogen Peroxide Assay kit (Molecular Probes, Invitrogen, Eugene, OR, USA). Briefly, H_2_O_2_ released from the treated cells reacted with the Amplex Red reagent containing horseradish peroxidase (HRP) to produce the red fluorescent oxidation product, resorufin. The fluorescence of resorufin was determined at 545 nm excitation and 590 nm emission using a fluorescence microplate reader (Molecular Devices Corp., Silicon Valley, CA, USA). The results were calculated as relative values to the control.

### Quantitative real-time polymerase chain reaction

Total RNA was extracted from treated VSMCs using Trizol (Invitrogen, Carlsbad, CA, USA) according to the manufacturer’s instructions. One microgram of total RNA was reverse transcribed to cDNA using PrimeScript cDNA Synthesis kit (TaKaRa Shuzo Co., Ltd, Otsu, Japan). The synthesized cDNA was used for quantitative polymerase chain reaction in the StepOne Plus Real-time PCR system (Applied Biosystems, Foster City, CA, USA) with SYBER Green PCR master mix (Life Technologies, Carlsbad, CA, USA). Transcript level of target genes was calculated by the 2^-ΔΔCT^ method. All primers used for qRT-PCR were designed using the Primer Express 3.0.1 software (Applied Biosystems, Foster City, CA, USA), and are as follows: *PiT-1* (Forward 5′-CCA GCA TAG ATA GCA CCG TGA AT-3′ and Reverse 5′-TGG CCA CTG GAG TTT ATT TGG-3′), *SM22α* (Forward 5´- GAG CAG GTG GCT CAG TTC CT-3´ and Reverse 5´-CTG CCA TGT CTT TGC CTT CA-3´), *α-SMA* (Forward 5´-TCC GGA GCG CAA ATA CTC TGT-3´ and Reverse-5´-CCG GCT TCA TCG TAT TCC TGT-3´), *CBFA1* (Forward 5´-TGC CAT TAT TGC TGC TGT GTT T-3´ and Reverse 5´-ACC CGC CAT GAC AGT AAC CA-3´), *OSX* (Forward 5´- GCC CTG CCA CAC CAA CA-3´ and Reverse 5´-AGG AAA TAA GCT TGA GAA GCA GAA A-3´), *E11* (Forward 5´-TCT GGT GGC AAC AAG TGT CAA-3´ and Reverse 5´-GGC GCT TGG ACT TTG TTC TT-3´), *DMP-1* (Forward 5´-AAG CAA GAA AGG ATC TGC ATG AT-3´ and Reverse-5´- ATA CAT GGA CAC CCA ATA GCT TTG3´), *SOST* (Forward 5′-ATC ACA TCC GCC CCA ACT T-3′ and Reverse 5′-CCT TTC ACT TCT CTT CGG AAG GT-3′), *FDZ3* (Forward 5′-CCC TCT GTA TTT TGG GTT GGA A-3′ and Reverse 5′-CCT GTC GGC TCT CAT TCA CTA TC-3′), *DKK-1* (Forward 5′-AGC ACC TTG GAT GGG TAT TCC-3′ and Reverse 5′-TGA CCG GAG ACA AAC AGA ACC T-3′), *NOX4* (Forward: 5`-CCT TCC GTT GGT TTG CAG AT-3′ and Reverse: 5`-GTT TGA CTG AGG TAC AGC TGG ATG T-3´), *p22*^*phox*^ (Forward 5´-ACT TTG GTG CCT ACT CCA TTG TG-3´ and Reverse 5´- TGT CCC CAG CGC TCC AT-3´), and *GAPDH* (Forward 5`-TTC ACC ACC ATG GAG AAG GCT-3’ and reverse 5`-TGG TTC ACA CCC ATG ACG AAC-3’).

### Western blot

After treatment, twenty micrograms of cellular extract were separated by 10% sodium dodecyl sulfate-polyacrylamide (SDS) gel electrophoresis and transferred to a nitrocellulose membrane. The membrane was blocked for 1h and incubated overnight at 4°C with primary antibodies against PiT-1 (1:3000, Abcam), PI3K p85 (1:1000, Cell signaling), phospho-PI3K p85 (Tyr458; 1:1000, Cell signaling), total AKT (1:1000, Cell signaling), phospho-AKT (ser473; 1:1000, Cell signaling), α-SMA (1:5000, Sigma-Aldrich), RUNX2 (1:1000, Abcam), FDZ3 (1:1000, Abcam), DKK-1 (1:1000, Abcam) or GAPDH (1:3000, Cell signaling). After washing, the membrane was incubated with horseradish peroxidase-conjugated secondary antibody (Dako, Glostrup, Denmark) for 1 h and detected using ECL reagents (Amersham Bioscience, Piscataway, NJ, USA). The intensity of the bands was quantified using the Scion Image software (Scion, Frederick, MD, USA).

### Immunohistochemistry

The paraffin-embedded tissues were sliced into three-micrometer sections, deparffinized, and endogenous peroxidase blocked by incubation with 0.3% hydrogen peroxide. After washing with PBS, the tissue samples were microwaved in 0.01M citrate buffer for antigen retrival, non-specific binding was blocked by treating with 5% skim milk for 30 min, and then incubated with primary antibodies against rabbit anti-RUNX2 antibody (Abcam) at 4°C overnight. After washing, the tissue samples were incubated with horseradish peroxidase-conjugated secondary antibody (DAKO, Glostrup, Denmark) and detected by the EnVision-HRP kit (Dako, Carprinteria, CA, USA). The immunolabeling was examined under a Leica DM IRB inverted microscope (Leica Microsystems, Wetzlar, Germany) equipped with a CoolSNAP HQ camera (Photometrics, Tucson, AZ, USA).

### Statistical analysis

Data are presented as the means ± standard error of the mean (SEM) and were repeated at least three independent experiments. All statistical analyses were performed by one-way analysis of variance (ANOVA) with Tukey’s *post hoc* analysis using GraphPad prism 5.01 programs (GraphPad Software Inc., La Jolla, CA, USA). P < 0.05 was considered statistically significant.

## Results

### Gemigliptin attenuates aortic calcification in adenine-induced CKD rats

**Fig 1:**
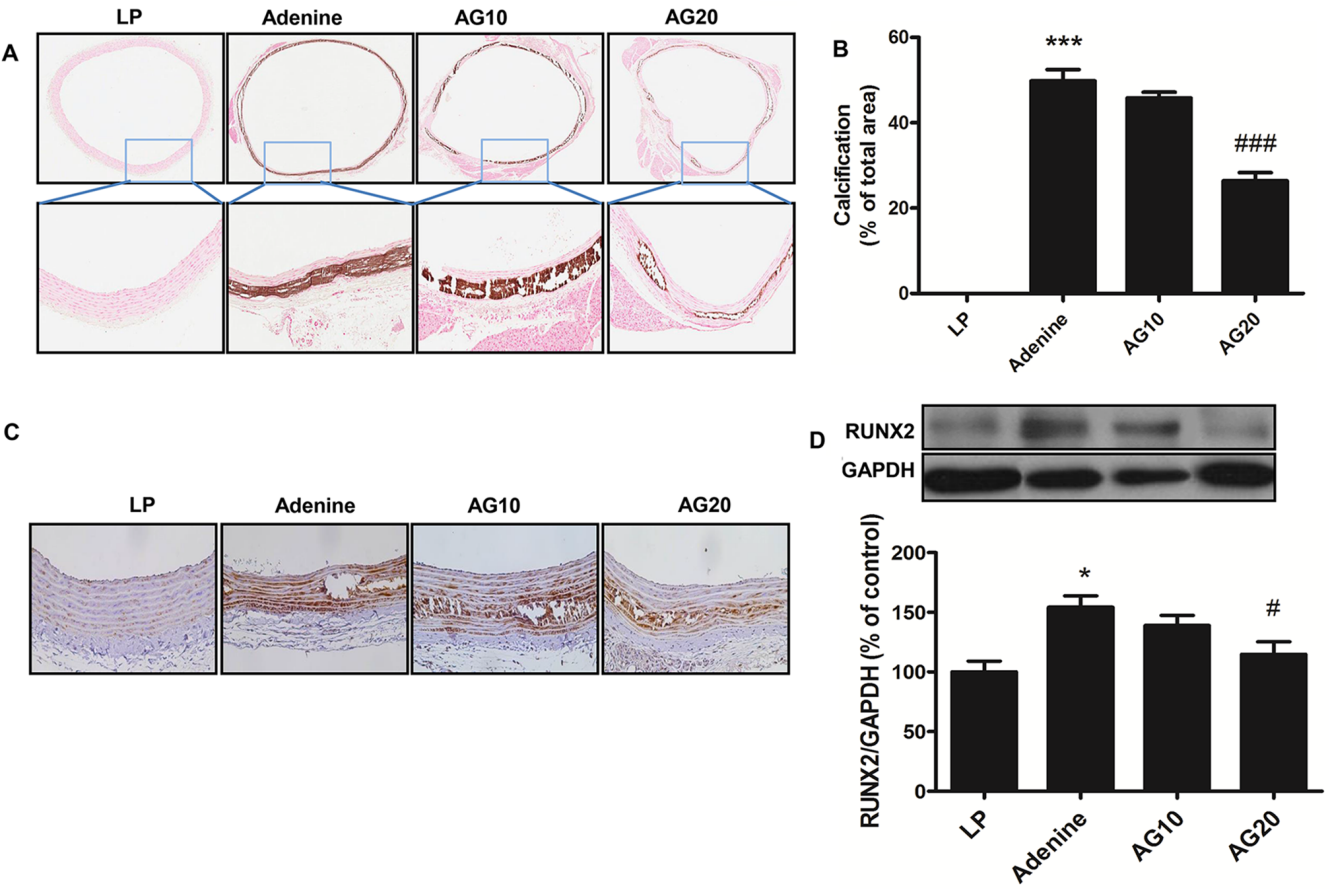
Gemigliptin attenuates vascular calcification in the abdominal aorta. (A) Representative photomicrographs of the abdominal aorta stained by Von Kossa staining (original magnification, × 17 and × 100). (B) The percentage of calcified area was calculated as the ratio of the Von Kossa positive area versus the total tissue area. (C) Representative photomicrographs of immunohistochemistry for RUNX2 (original magnification, × 200) are shown. (D) Representative immunoblot images and the quantification of RUNX2 expression are shown. GAPDH was used as the loading control. The results are expressed as percentage of control. Results are presented as mean ± SEM (n = 5 for LP, low protein group; n = 5 for adenine group; n = 6 for AG10, adenine-gemigliptin [10mg/kg] group; n = 6 for AG20; adenine-gemigliptin [20mg/kg] group). RUNX2, runt-related transcription factor-2. *P<0.5, **P<0.01, and ***P<0.001 compared with LP group, and #P<0.5, ###P<0.001 compared with adenine group.

Rats fed adenine for 4 weeks showed significant reduction in food intake and body weight. Serum blood urea nitrogen (BUN) and creatinine levels significantly increased in adenine-fed rats compared to the control group, which confirmed the establishment of a CKD model. Serum phosphate significantly increased but serum calcium was not changed (Table 1). In addition, adenine-fed rats showed a remarkable increase in calcium deposition in the media layer of the abdominal aorta compared to that observed for the control group, whereas calcium deposition was significantly reduced by a high dose (20 mg/kg) of gemigliptin (Fig 1A and 1B). Furthermore, we investigated the change of RUNX2 expression using immunohistochemistry (Fig 1C) and western blot analysis (Fig 1D). Increased RUNX2 expression in adenine group was significantly reduced by gemigliptin treatment.

**Table 1:** Body weight, food intake, and biochemical parameters in an animal model of chronic kidney disease.

	LP	Adenine	AG10	AG20
Body weight (g)	400±6.83	244±13.50*	247±5.41*	261±10.91*
Food intake (g/day)	25.8±0.69	7.2±1.28*	6.7±1.04*	6.7±1.09*
BUN (mg/dL)	8.42±0.96	183.63±45.05*	107.0±14.26*^#^	113.08±22.40*^#^
Creatinine (mg/dL)	0.44±0.03	3.35±0.48*	2.95±0.37*	2.90±0.51*
Calcium (mg/dL)	9.44±0.09	9.13±0.58	9.45±0.42	9.22±0.21
Phosphate (mg/dL)	5.66±0.36	21.73±2.26*	19.73±1.90*	19.24±1.14*
Glucose (mg/dL)	118±9.9	110±17.2	110±13.5	90.4±8.2

LP, low protein; AG10, adenine-gemigliptin (10 mg/kg) group; AG20, adenine-gemigliptin (20mg/kg) group; and BUN, blood urea nitrogen.

*P<0.001 compared with low protein group and

#P<0.001 compared with adenine group.

### Gemigliptin attenuates high phosphate-induced mineralization in VSMCs

**Fig 2:**
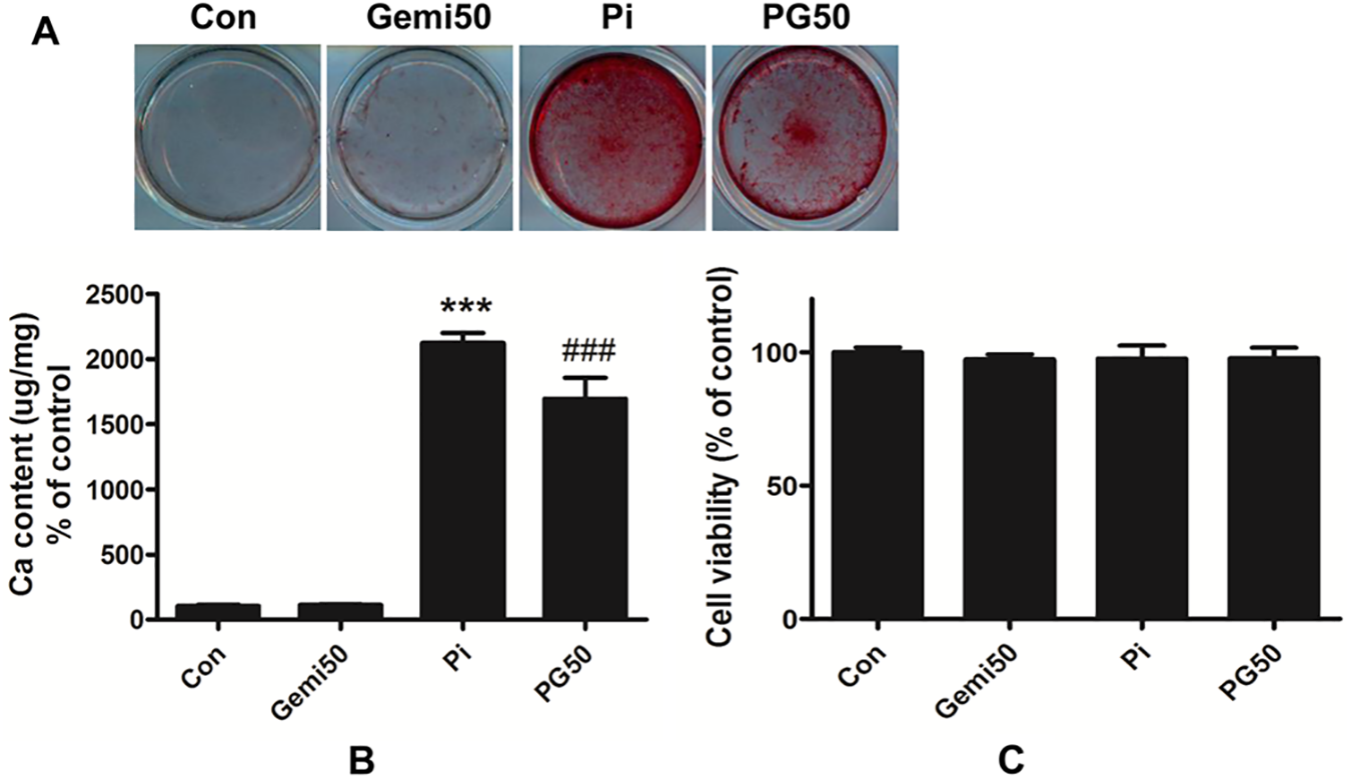
Gemigliptin attenuates high phosphate-induced vascular calcification in VSMCs. (A) Visualization of calcium deposition on VSMCs was assessed by alizarin red staining. Representative pictures were shown. (B) After HCl decalcification, calcium content of the cells was assayed. The value was normalized by the total protein and expressed as percentage of control. (C) Cell viability was determined using MTT assay. The results are expressed as percentage of control. Results are presented as mean ± SEM (n = 6–7 in each group). Con, control; Gemi50, 50 μM gemigliptin; Pi, 3mM phosphate; PG50, 3 mM phosphate and 50 μM gemigliptin. ***P<0.001 compared with control and ###P<0.001 compared with phosphate group.

To investigate whether gemigliptin attenuates high phosphate-induced VC in cultured VSMCs, we quantified the calcium content after HCl decalcification and visualized calcium deposition using alizarin red staining after treatment with 3 mM phosphate and/or 50 μM gemigliptin for 14 days. VSMCs treated with high phosphate showed stronger red staining (Fig 2A) and significantly increased calcium content (Fig 2B) than the control group. However, VSMCs treated with phosphate and gemigliptin showed weaker staining (Fig 2A) and decreased calcium content (Fig 2B) than phosphate group. Additionally, we investigated the cytotoxic effect of 50 μM gemigliptin and/or 3 mM phosphate on VSMCs using an MTT assay. Treatments with gemigliptin and/or phosphate did not affect cell viability (Fig 2C). Therefore, these results suggest that gemigliptin attenuates high phosphate-induced VC without decreasing cell viability.

### Gemigliptin attenuates type III sodium-dependent phosphate cotransporter PiT-1 expression

**Fig 3:**
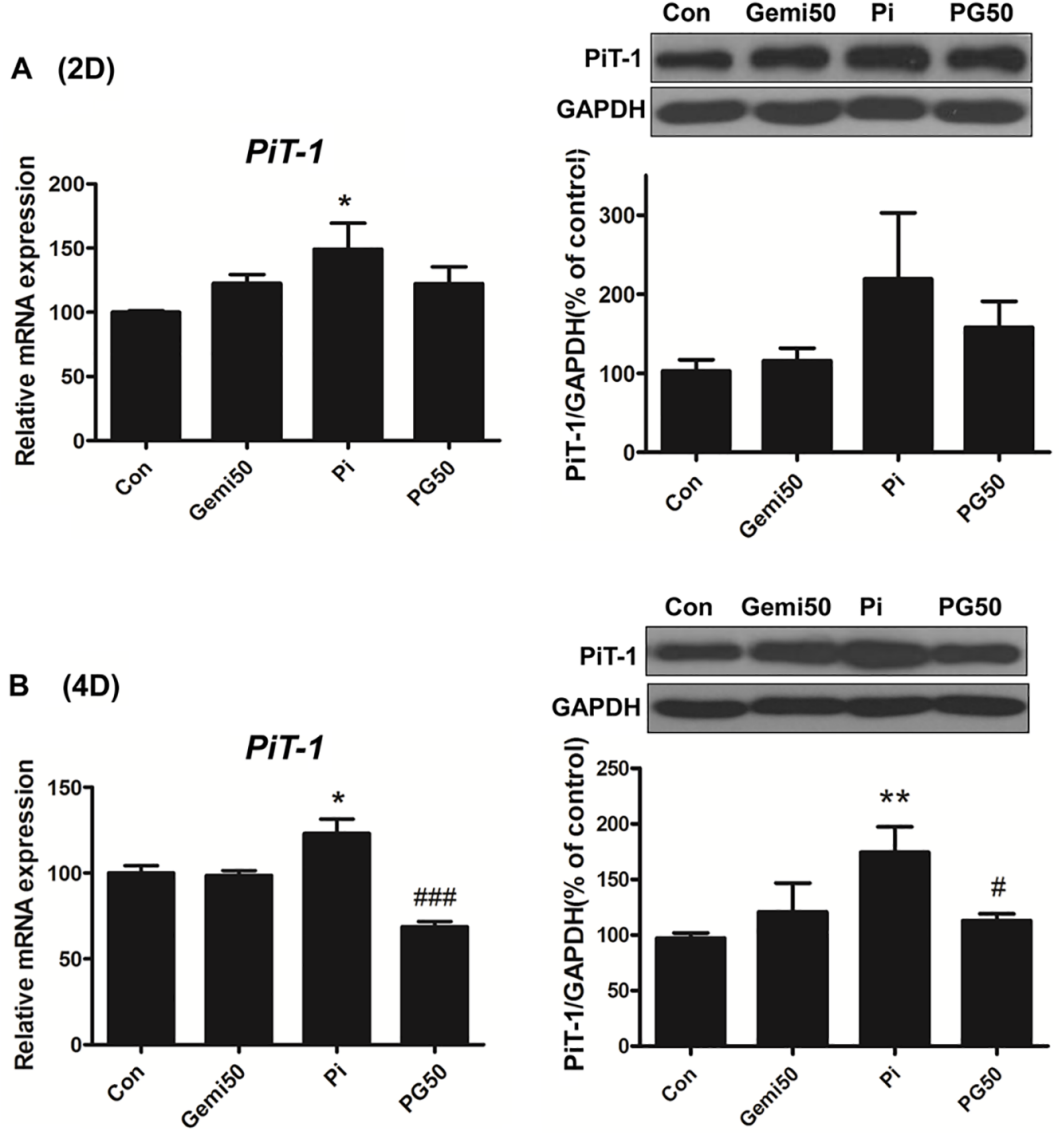
Gemigliptin attenuates the expression of sodium phosphate co-transporter PiT-1. The expression of PiT-1 was evaluated by qRT-PCR and western blot after treatment with 3 mM phosphate and/or 50 μM gemigliptin for 2 days (A) or 4 days (B). GAPDH was used as the loading control. The results are expressed as percentage of control. Results are presented as mean ± SEM (n = 5–6 in each group). Con, control; Gemi50, 50 μM gemigliptin; Pi, 3mM phosphate; PG50, 3mM phosphate and 50 μM gemigliptin. *P<0.5, **P<0.01, ***P<0.001 compared with control and #P<0.5, ##P<0.01, ###P<0.001 compared with phosphate group.

Phosphate is taken into the cell through type III sodium-dependent phosphate transporters (PiT-1 and PiT-2) [24]. Among the transporters, PiT-1 is predominantly expressed in human VSMCs [25]. To investigate whether gemigliptin affects PiT-1 expression, we treated VSMCs with phosphate and/or gemigliptin for 2 or 4 days. High phosphate significantly increased mRNA expression and protein level of the type III sodium-dependent phosphate transporter PiT-1 at 2 days (Fig 3A) and 4 days (Fig 3B). Gemigliptin attenuated phosphate-induced PiT-1 expression at 4 days. These results might be suggested that gemigliptin is associated with reduction of intracellular phosphate uptake through decreased expression of PiT-1.

### Gemigliptin modulates ROS generation in VSMCs

**Fig 4:**
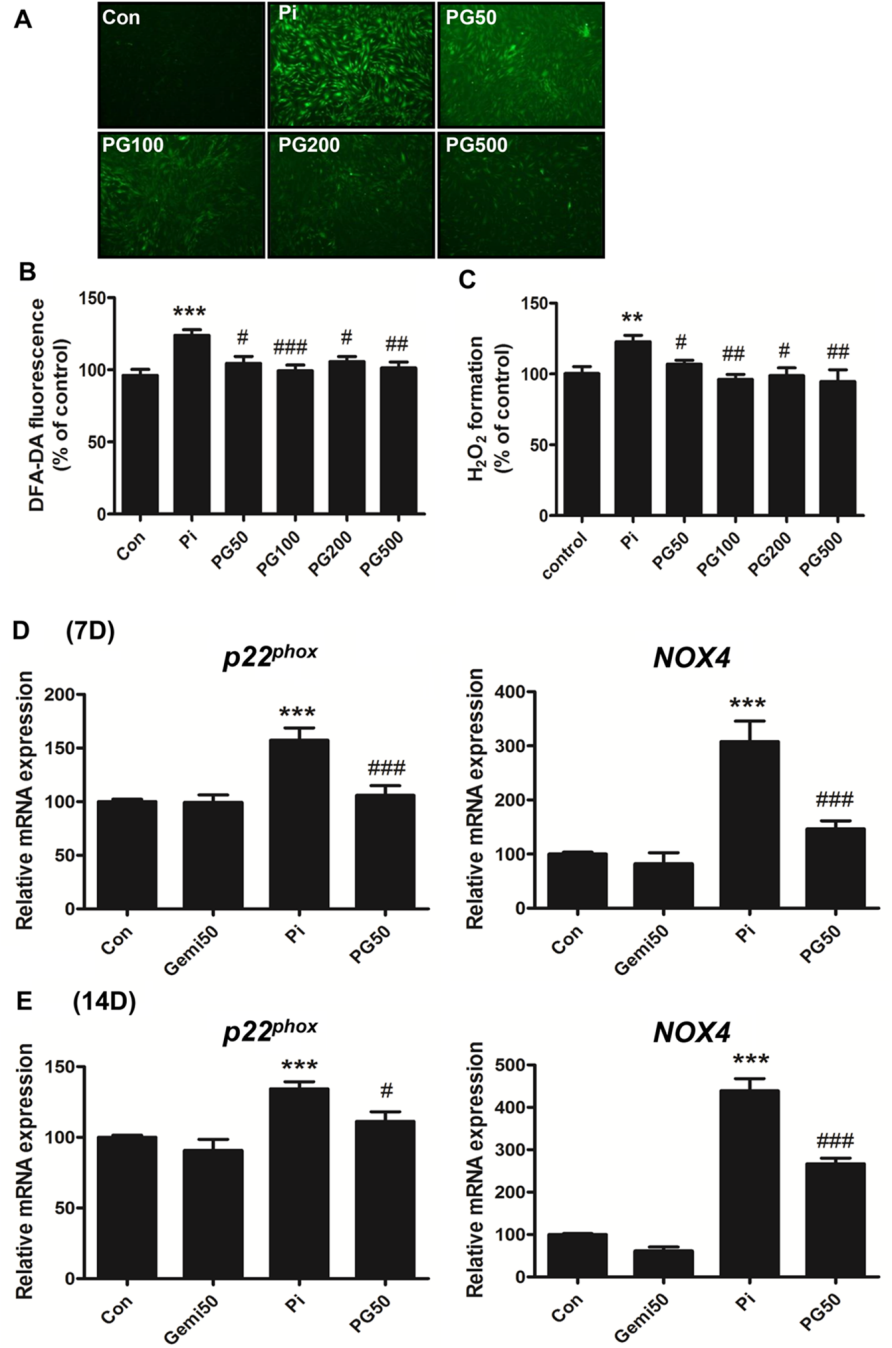
Gemigliptin modulates ROS generation and the expression of NADPH oxidase *NOX4* and NADPH oxidase subunit *p22*^*phox*^. (A) Intracellular ROS generation was visualized by fluorescence microscopy, and (B) quantified in the cellular lysate after staining with 10 μM H_2_DCF-DA. (C) The level of H_2_O_2_ generation was measured using the Amplex red hydrogen peroxide assay kit. The mRNA expression of *NOX4* and *p22*^*phox*^ was analyzed by qRT-PCR after treating VSMCs with phosphate and/or gemigliptin for (D) 7 days and (E) 14 days. The results are expressed as percentage of control. Results are presented as mean ± SEM (n = 6–7 in each group). Con, control; Pi, 3 mM phosphate; PG50 –PG500, 3 mM phosphate and 50 μM– 500 μM gemigliptin. *P<0.5, **P<0.01, ***P<0.001 compared with control and #P<0.5, ##P<0.01, ###P<0.001 compared with phosphate group.

NADPH oxidase (NOX) is an important source of ROS generation in vascular tissue [26]. NOX-derived ROS generation plays an important role in VC [27]. To investigate whether gemigliptin affects high phosphate-induced ROS generation, we compared intracellular ROS using DCF-DA staining and by direct measurement of H_2_O_2_ generation between the phosphate-treated group and the group co-treated with phosphate and gemigliptin. Phosphate significantly increased intracellular ROS (Fig 4A and 4B) and H_2_O_2_ generation (Fig 4C), whereas gemigliptin decreased ROS generation at 2 h (Fig 4A–4C). In addition, we investigated the expression of *NOX4* and NADPH oxidase subunit *p22*^*phox*^ at 7 and 14 days, respectively. *NOX4* and *p22*^*phox*^ mRNA were increased in the high phosphate-treated VSMCs, whereas they were significantly attenuated by gemigliptin treatment (Fig 4D and 4E)

### Gemigliptin reduces high phosphate-induced phospho-PI3K/AKT signaling

**Fig 5:**
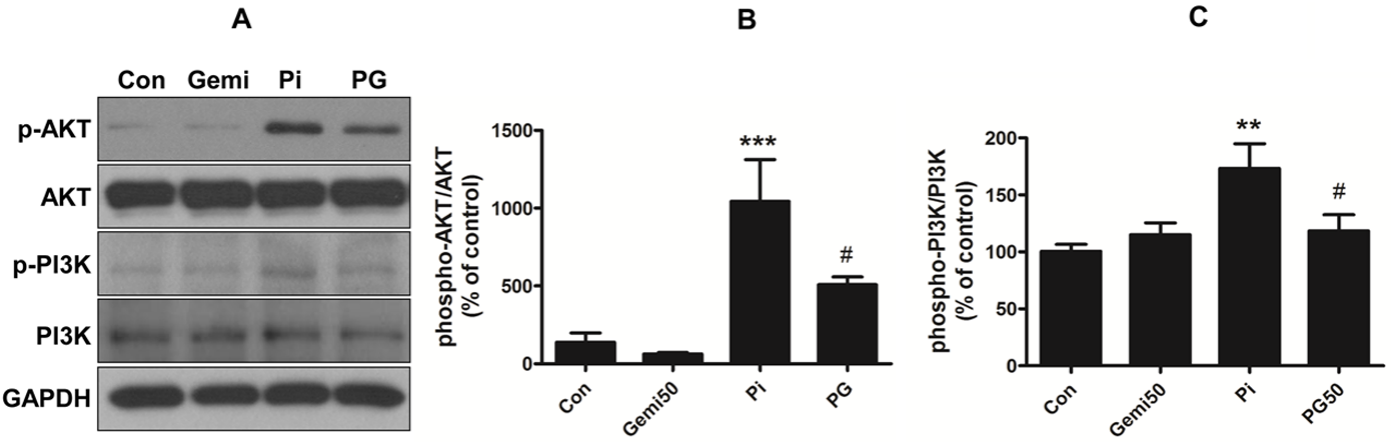
Gemigliptin attenuates phosphate-induced phospho-PI3K-AKT signaling pathway. (A) VSMCs were treated with 3 mM phosphate and/or 50 μM gemigliptin for 24 h. Representative immunoblot images are shown. Graph represents quantitative data as (B) ratio of phospho-AKT/total-AKT or (C) phospho-PI3K/total PI3K. The results are expressed as percentage of control. Results are presented as mean ± SEM (n = 4–5 in each group). Con, control; Gemi50, 50 μM gemigliptin; Pi, 3 mM phosphate; PG50, 3 mM phosphate and 50 μM gemigliptin. *P<0.5, **P<0.01, ***P<0.001 compared with control, #P<0.5, ##P<0.01, ###P<0.001 compared with phosphate group.

We investigated whether high phosphate-induced ROS generation activates the PI3K/AKT intracellular signaling pathway. Using VSMCs treated with phosphate and/or gemigliptin for 24 h, phosphorylation of PI3K and AKT was measured by western blot. Phosphate significantly induced phosphorylation of PI3K p85 and AKT (Fig 5), whereas they significantly decreased in VSMCs co-treated with phosphate and gemigliptin. No changes in total PI3K and AKT were observed in all groups (Fig 5). These results indicate that gemigliptin attenuates VC in VSMCs through inhibition of PI3K/AKT phosphorylation.

### Gemigliptin attenuates high phosphate-induced Wnt signaling

**Fig 6:**
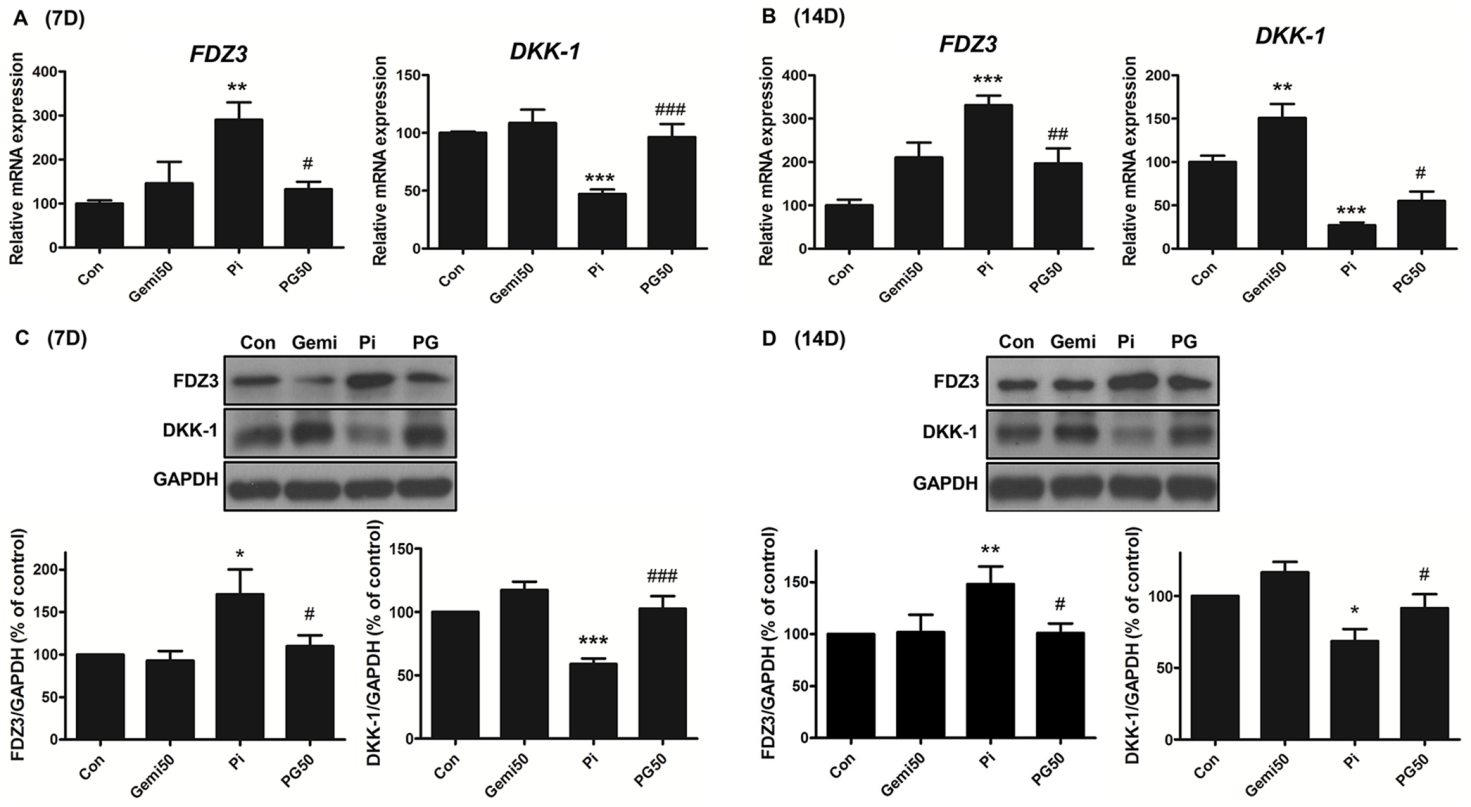
Gemigliptin attenuates high phosphate-induced Wnt signaling. The expression of *FDZ3* and *DKK-*1 was evaluated by qRT-PCR (A and B) and western blot (C and D) in VSMCs after incubating with 3 mM phosphate and/or 50 μM gemigliptin for 7 days (A and C) and 14 days (B and D). The results are expressed as percentage of control. Results are presented as mean ± SEM (n = 5–6 in each group). FDZ3, frizzled-3; DKK-1, dickkopf-1; Con, control; Gemi50, 50 μM gemigliptin; Pi, 3mM phosphate; PG50, 3mM phosphate and 50 μM gemigliptin. *P<0.5, **P<0.01, ***P<0.001 compared with control and #P<0.5, ##P<0.01, ###P<0.001 compared with phosphate group.

To confirm the effect of gemigliptin treatment on the Wnt signaling pathway, a key pathway affected in VC, we investigated the expression of frizzled-3 (FDZ3) and dickkopf-related protein-1 (DKK-1) after treatment with 3 mM phosphate and/or 50 μM gemigliptin for 7 and 14 days. Treatment with phosphate and gemigliptin restored the increased expression of FDZ3, a receptor for Wnt ligands, and downregulated expression of *DKK-1*, an antagonist of the Wnt pathway, induced by high phosphate at 7 (Fig 6A and 6C) and 14 days (Fig 6B and 6D). These results indicate that gemigliptin reduces VC through inhibition of the Wnt/β-catenin pathway, by decreasing FDZ3 expression and increasing DKK-1 expression.

### Gemigliptin reverses high phosphate-induced osteogenic trans-differentiation of VSMCs

**Fig 7:**
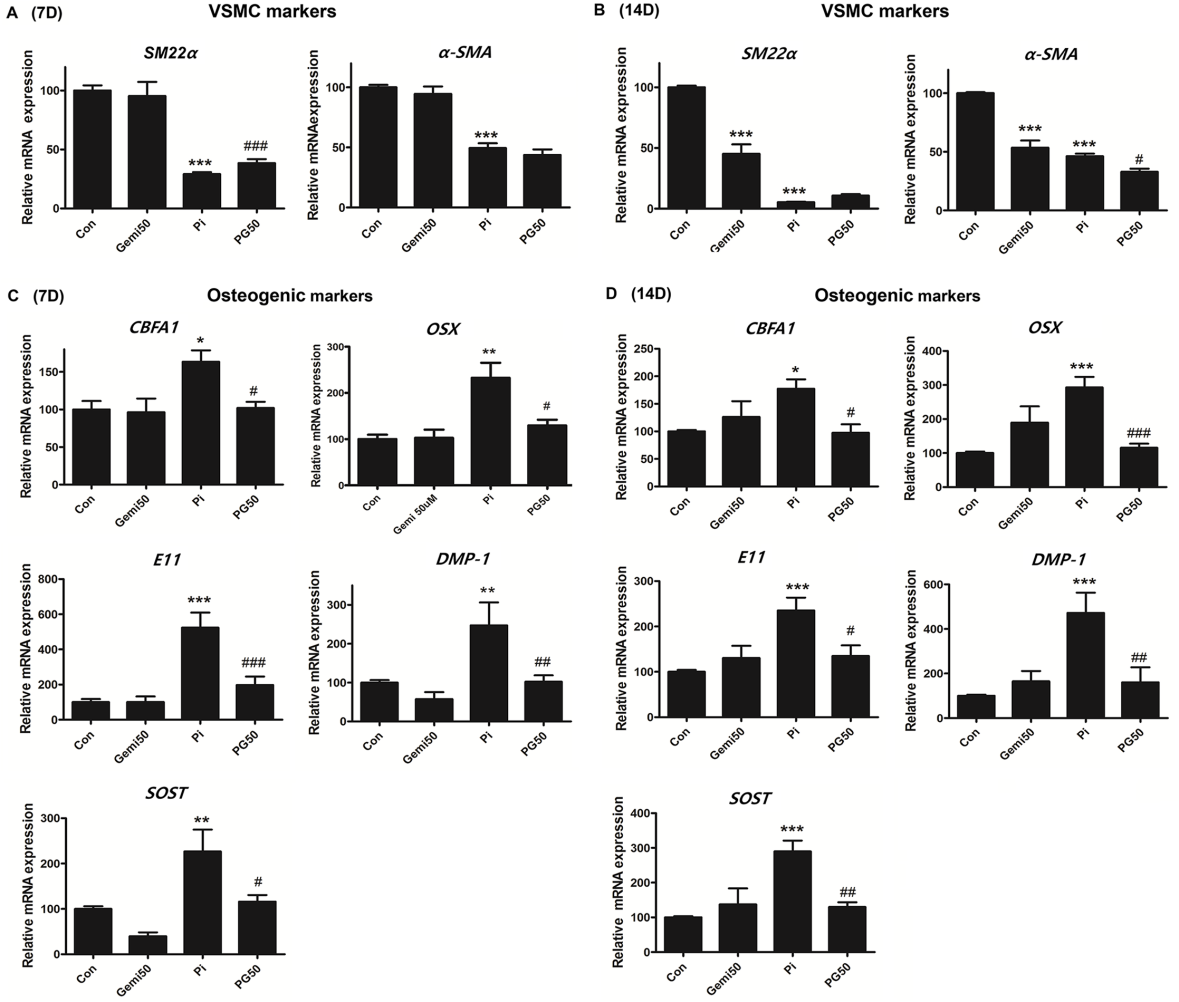
Gemigliptin attenuates high phosphate-induced osteogenic differentiation and restores VSMC markers. The mRNA expression of VSMC markers (*α-SMA* and *SM22α*) and osteogenic markers (*CBFA1*, *OSX*, *E11*, *DMP-1*, and *SOST*) were evaluated by qRT-PCR in cultured VSMCs after incubating with 3 mM high phosphate and/or 50 μM gemigliptin for 7 days (A and C) and 14 days (B and D). The results are expressed as percentage of control. Results are presented as mean ± SEM (n = 6–7 in each group). CBFA1, core-binding factor alpha a; OSX, osterix; OC, osteocalcin; SOST, sclerostin, Con, control; Gemi50, 50 μM gemigliptin; Pi, 3mM phosphate; PG50, 3 mM phosphate and 50 μM gemigliptin. *P<0.5, **P<0.01, ***P<0.001 compared with control and #P<0.5, ##P<0.01, ###P<0.001 compared with phosphate.

To confirm whether gemigliptin regulates high phosphate-induced osteogenic trans-differentiation on VSMCs, we examined the change of cell phenotype by measuring VSMC markers (*SM 22α* and *α-SMA*) and osteogenic markers (*CBFA1*, *DMP-1*, *OSX*, *SOST*, and *E11*) using quantitative real-time-polymerase chain reaction (qRT-PCR) following treatment for 7 and 14 days. High phosphate significantly decreased the expression of VSMC markers (Fig 7A and 7B) and induced the expression of osteogenic markers at 7 and 14 days (Fig 7C and 7D), respectively. However, gemigliptin significantly restored the reduced *SM22α* expression at 7 days (Fig 7A) and attenuated the induction of osteogenic markers at 7 and 14 days by high phosphate exposure (Fig 7C and 7D). These results indicate that gemigliptin reverses the high phosphate-induced cellular phenotype, such as osteogenic trans-differentiation of VSMCs.

## Discussion

In this study, for the first time, we investigated the protective effect of gemigliptin against VC in an adenine-induced CKD model and in cultured VSMCs. Using a rat model of adenine-induced CKD, we demonstrated an increased VC in abdominal aortic wall. Gemigliptin treatment significantly reduced VC and attenuated the expression of the osteogenic marker, RUNX2. The protective effect of gemigliptin on VC was independent of the level of serum phosphorus. Similarly, a previous report showed that the protective effect of diosgenin against VC depends on the direct action on VSMCs regardless of blood chemistry [28].

**Fig 8:**
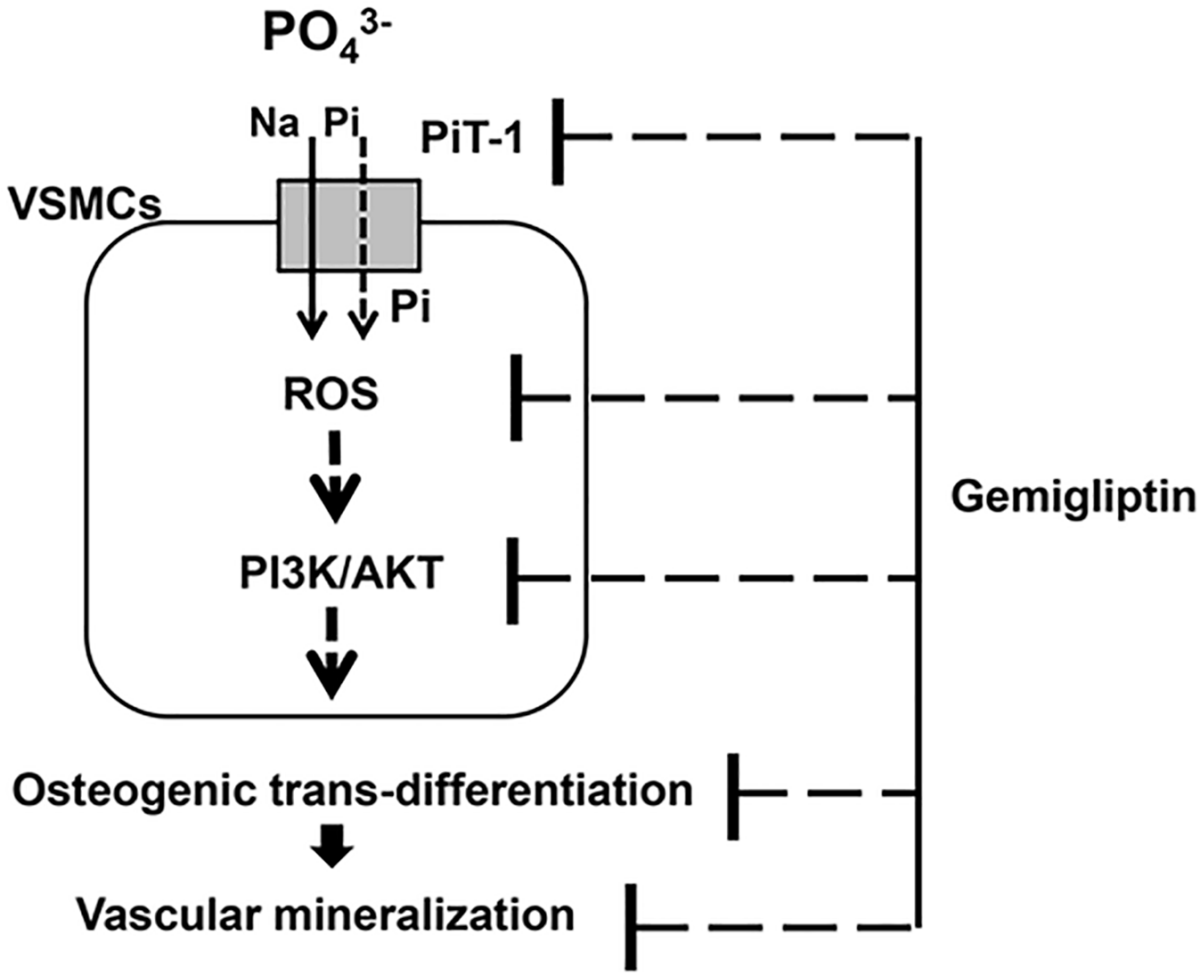
A flow diagram of the effect of gemigliptin against high-phosphate-induced vascular calcification. Gemigliptin attenuated vascular calcification and osteogenic trans-differentiation in VSMCs via multiple steps including downregulation of PiT-l expression, and suppression of ROS generation, phosphor-PI3K/AKT, and Wnt signaling pathway.

Based on the *in vivo* results, we proceeded on to an *in vitro* experiment using cultured VCMCs to explore the mechanisms of gemigliptin against VC. As a result, the DPP-4 inhibitor, gemigliptin attenuated high phosphate-induced VC in cultured VSMCs via the following mechanisms. Gemigliptin reduced intracellular phosphate uptake by downregulating the expression of the type III sodium phosphate co-transporter PiT-1 and attenuated oxidative stress by reducing ROS generation and mRNA expression of NADPH oxidase (*NOX4*, *p22*^*phox*^ subunit). Therefore, gemigliptin attenuated the PI3K/AKT signaling pathway and modulated Wnt signaling. As a result, gemigliptin attenuated osteogenic trans-differentiation of VSMCs and resulted in reduction of VC (Fig 8).

Gemigliptin, a novel DPP-4 inhibitor was approved for treatment of patients with type 2 DM in June 2012 in Korea. Compared to other DPP-4 inhibitors (sitagliptin and vildagliptin), it has a long-acting DPP-4 inhibition effect with strong and highly selective binding properties [20]. Gemigliptin is suggested to have a cardiovascular protective effect in addition to glucose-lowering effect similar to sitagliptin, vildagliptin, saxagliptin, linagliptin, and alogliptin, which are the commercially available DPP-4 inhibitors thus far. Previous studies reported that gemigliptin inhibited endoplasmic reticulum-induced apoptosis and inflammation in H9c2 cardiomyocytes through the Akt/PERK/CHOP and IRE1α/JNK-p38 pathways [29]. In addition, gemigliptin reduced lipopolysaccharide (LPS)-induced expression of adhesion molecules and pro-inflammatory cytokines (VCAM-1, E-selectin, TNF-α, MCP-1, IL-1ß, and IL-6) in HUVECs and inhibited LPS- and LDL-induced foam cell formation in macrophage-like THP-1 cells. These anti-inflammatory and anti-atherosclerotic effects of gemigliptin were mediated by attenuating NF-κB and JNK signaling through Akt/AMPK-dependent mechanisms [21]. However, to the best of our knowledge, this is the first study to investigate the effect of the DPP-4 inhibitor, gemigliptin on high phosphate-induced VC.

High phosphate is a key factor of arterial medial calcification. Suggested underlying mechanisms of phosphate-induced VC are as follows: osteoblastic phenotype change of VSMCs, VSMC apoptosis, loss of inhibitors, extracellular matrix degradation, and release of matrix vesicles [30–32]. The type III sodium-dependent phosphate transporter PiT-1 is more predominantly expressed in human VSMCs [24, 25]. Phosphate uptake through PiT-1 plays an important role in the regulation of mineralization and osteogenic differentiation *in vitro* and *in vivo* [33]. In this study, high phosphate induced the expression of PiT-1 at 2 and 4 days, whereas these were significantly downregulated by gemigliptin at 4 days. This may be suggested that gemigliptin decreases intracellular phosphate uptake by downregulating the expression of the sodium phosphate transporter PiT-1. A previous report has shown that the change in PiT-1 expression is associated with osteogenic trans-differentiation. Knockdown of *PiT-1* by small interfering RNAs attenuated phosphate uptake as well as phosphate-induced phenotypic transition and SMC calcification *in vitro* [34, 35]. Furthermore, inhibition of the phosphate transporter by phosphonoformic acid (PFA) decreased the expression of CBFA1, osteopontin (OPN), and osteocalcin (OC) and subsequently inhibited elevated SMC calcification. Therefore, PiT-1 may have an important role in high phosphate-induced VC. In addition, increased intracellular phosphate through PiT-1 transporter regulates mitochondrial membrane potential (MP) and ROS production [3]. ROS could be produced either from mitochondria or by superoxide-producing enzymes such as NADPH oxidase, xanthine oxidase, and cytochrome P450 [36]. A previous report has shown that oxidative stress by phosphate induced osteogenic trans-differentiation and VC [3]. Furthermore, it is reported that the major source of ROS generation in VSMCs is NADPH oxidase that produces intracellular superoxide via p22^phox^ and NOX4 [26]. Nox4-derived ROS are important to the maintenance of the transdifferentiated phenotype of VMSCs and associate with smooth muscle transdifferentiation markers [37]. In addition, the NADPH oxidase subunit p22^phox^ and NOX2 were observed around calcifying foci [26, 38]. In this study, we investigated whether gemigliptin attenuates ROS generation such as H_2_O_2_ in high phosphate environments since biologically, superoxide generated by NADPH oxidase is short-lived and is rapidly reduced to H_2_O_2_ by superoxide dismutase. As a result, high phosphate significantly increased intracellular ROS, particularly H_2_O_2_ production, in cultured VSMCs, which was attenuated by gemigliptin. We also investigated the expression of NADPH oxidase as a ROS generator in VSMCs. Phosphate increased the expression of *NOX4* and *p22*^*phox*^, whereas their expression was decreased by gemigliptin at 7 and 14 days. It is known that suppression of *NOX4* mRNA expression markedly decreases in NADPH oxidase-dependent superoxide production [26]. Previous studies have suggested possible links between ROS and osteogenic trans-differentiation and calcification; H_2_O_2_ induces trans-differentiation of VSMCs into osteoblastic cells through activation of RUNX2, a key transcriptional factor, and causes calcium mineral deposition [39, 40]. Indoxyl sulfate (IS) stimulates ROS generation by upregulating NOX4 and induces the expression of osteoblastic markers such as CBFA1, ALP, and osteopontin in VSMCs. Inhibition of IS-induced ROS generation by an NADPH oxidase inhibitor and antioxidant attenuates osteogenic trans-differentiation [41]. In addition, the antioxidant tempol decreases calcium deposition by blocking the trans-differentiation into osteoblastic cells [42]. Hydrogen peroxide stimulates tyrosine phosphorylation of PI3K [43]. Phosphorylation of PI3K and its downstream AKT plays an important role in osteoblast trans-differentiation [44]. In this study, we investigated whether gemigliptin inhibits PI3K/AKT phosphorylation through reduction of high phosphate-induced oxidative stress. As a result, phosphate increased ROS and phosphorylation of PI3K and AKT, which were significantly decreased by gemigliptin. This result suggested that high phosphate-induced ROS generation activates PI3K/AKT, which is attenuated by gemigliptin. In this study, we also investigated that the association of gemigliptin-induced reduction in ROS and phospho-PI3K/AKT and phenotype changes of VSMCs. As a result, gemigliptin attenuated the expression of osteogenic markers by high phosphate in VSMCs. Previous studies suggested that BMP-2 stimulated PI3K/AKT activity in osteogenic cells and inhibition of PI3K/AKT activity blocked BMP-2-induced alkaline phosphatase, an early marker of osteoblastic induction [44]. Additionally, inhibition of hydrogen peroxide-induced AKT signaling blocked VSMC calcification and RUNX2 induction [40]. Therefore, RUNX2 induction by AKT activation has an important role in oxidative stress-induced VSMC calcification [3, 40]. Recent studies demonstrated that the GLP-1 analog liraglutide attenuates the osteoblastic trans-differentiation and calcification through inhibition of PI3K/AKT signaling in VSMCs [45]. Another DPP-4 inhibitor decreases ROS generation in indoxyl sulfate (IS)-treated HK-2 cells and has anti-apoptotic activity to reduce IS-induced renal damage by regulating the ROS/p38MAPK/ERK and PI3K-AKT pathways [46].

As a next step, we measured the change in Wnt/ß-catenin pathway in high phosphate-induced VC and after gemigliptin treatment. The Wnt/β-catenin pathway has known to play a key role in VC by inducing the expression of osteogenic markers through the upregulation of RUNX2 transcription. Namely, high phosphate induces WNT secretion, activates β-catenin via stabilization of β-catenin and inhibition of GSK-3β or WNT-2A protein, and then upregulates RUNX2 expression [7, 47, 48]. We evaluated the expression of *FDZ3* (a receptor for Wnt ligands) and *DKK-1* (an antagonist of the Wnt pathway) in the Wnt pathway. Gemigliptin reversed the high phosphate-induced *FDZ3* expression and reduced the expression of *DKK-1* in this study. In other words, gemigliptin attenuated the high phosphate-induced osteogenic trans-differentiation and calcium accumulation in VSMCs by inhibition of the Wnt signaling pathway through downregulation of *FDZ3* expression and upregulation of *DKK-1* expression. Consistent with this, previous studies have shown that phosphate-induced calcification is decreased by co-treatment of DKK-1 with high phosphate [49]. In addition, magnesium reduces calcification and osteogenic differentiation by reducing the expression of *FDZ3*, VCAN/versican, cyclin D1, and c-Myc and enhancing DKK-1 expression in VSMCs exposed to high phosphate [8].

In addition, high phosphate induces changes in cellular phenotypes by altering the expression of osteogenic markers such as *CBFA1*, *OSX*, *E11*, *DMP-1*, and *SOST* and vascular smooth cell markers such as *α-SMA* and *SM22α* in cultured VSMCs. Gemigliptin attenuated VSMC mineralization and restored the changes in osteogenic markers and VSMC markers. The cytotoxic effect of gemigliptin and/or phosphate was evaluated using the MTT assay to determine whether the decrease in calcium content is confounded by cellular death, and we found that gemigliptin and/or phosphate did not change cellular viability.

In conclusion, gemigliptin attenuated VC *in vitro* and *in vivo* and osteogenic trans-differentiation of VSMCs by reducing PiT-1 expression, attenuating phosphate-induced oxidative stress, phospho-AKT/PI3K signaling, and Wnt signaling. Finally, the phenotypic changes of VSMCs induced by high phosphate were restored by gemigliptin treatment. Based on our data, we suggest gemigliptin might have a protective effect against VC. However, further studies are needed to confirm our findings, particularly clinical studies.
